# *De novo* identification of complex traits associated with asthma

**DOI:** 10.3389/fimmu.2023.1231492

**Published:** 2023-08-23

**Authors:** Roan E. Zaied, Tayaza Fadason, Justin M. O’Sullivan

**Affiliations:** ^1^ The Liggins Institute, The University of Auckland, Auckland, New Zealand; ^2^ The Maurice Wilkins Centre, The University of Auckland, Auckland, New Zealand; ^3^ Garvan Institute of Medical Research, Sydney, NSW, Australia; ^4^ Medical Research Council (MRC) Lifecourse Epidemiology Unit, University of Southampton, Southampton, United Kingdom; ^5^ Singapore Institute for Clinical Sciences, Agency for Science Technology and Research, Singapore, Singapore

**Keywords:** asthma, SNP function, comorbidity, expression quantitative trait loci, network analysis, gene regulation

## Abstract

**Introduction:**

Asthma is a heterogeneous inflammatory disease often associated with other complex phenotypes. Identifying asthma-associated diseases and uncovering the molecular mechanisms mediating their interaction can help detangle the heterogeneity of asthma. Network analysis is a powerful approach for untangling such inter-disease relationships.

**Methods:**

Here, we integrated information on physical contacts between common single nucleotide polymorphisms (SNPs) and gene expression with expression quantitative trait loci (eQTL) data from the lung and whole blood to construct two tissue-specific spatial gene regulatory networks (GRN). We then located the asthma GRN (level 0) within each tissue-specific GRN by identifying the genes that are functionally affected by asthma-associated spatial eQTLs. Curated protein interaction partners were subsequently identified up to four edges or levels away from the asthma GRN. The eQTLs spatially regulating genes on levels 0–4 were queried against the GWAS Catalog to identify the traits enriched (hypergeometric test; FDR ≤ 0.05) in each level.

**Results:**

We identified 80 and 82 traits significantly enriched in the lung and blood GRNs, respectively. All identified traits were previously reported to be comorbid or associated (positively or negatively) with asthma (e.g., depressive symptoms and lung cancer), except 8 traits whose association with asthma is yet to be confirmed (e.g., reticulocyte count). Our analysis additionally pinpoints the variants and genes that link asthma to the identified asthma-associated traits, a subset of which was replicated in a comorbidity analysis using health records of 26,781 asthma patients in New Zealand.

**Discussion:**

Our discovery approach identifies enriched traits in the regulatory space proximal to asthma, in the tissue of interest, without a priori selection of the interacting traits. The predictions it makes expand our understanding of possible shared molecular interactions and therapeutic targets for asthma, where no cure is currently available.

## Introduction

Asthma is a heterogenous phenotype with a wide range of interactions and presentations with other phenotypes (1). Its comorbidity with other complex diseases (e.g., eczema, mood disorders, and diabetes) suggests the existence of shared molecular pathways (2–5).

The genetics of asthma is complex, and the functional impacts of known asthma-associated variants are poorly understood (6). This is possibly due to data conglomeration issues arising from phenotyping complications due to the interaction of asthma with other complex traits. Despite this, the Trans-National Asthma Genetic Consortium (TAGC) performed a meta-analysis of genome-wide association studies (GWAS) and identified 878 SNPs in 18 loci as being associated with asthma (7). These loci were enriched for enhancer marks, particularly in immune cell types, suggesting roles in immune regulation (7). Yet, the specific causal mechanisms of these disease-associated SNPs (da-SNPs) and their contribution to the observed asthma comorbidity are largely yet to be elucidated (8). Identifying the regulatory connections that link asthma and asthma-associated conditions (both positively and negatively associated) will improve our understanding of inter-trait relationships and significantly impact our ability to apply and develop novel therapeutic approaches.

Assigning functions to da-SNPs is complex because most da-SNPs fall in non-coding regions and have small individual effect sizes (8). Moreover, expression quantitative trait loci (eQTL) analyses have identified long-range regulatory interactions at numerous loci, reducing the effectiveness of conventional nearest-gene approaches for identifying da-SNP functional targets (9–11). Spatially constrained gene regulatory networks integrate chromatin interaction (Hi-C) data and eQTL analyses to associate variants with the genes they regulate. These networks represent one approach for assigning functions to da-SNPs (12, 13).

The understanding that biologically similar traits and diseases segregate in modules within the same network neighborhood has been incorporated into attempts to identify conditions that are associated with asthma (14, 15). *A priori* selection of the interacting conditions enabled the identification of 1) overlapping genes between asthma and nine immune-mediated diseases (14); and 2) coding genes that link diseases with recognized similarities (e.g., chronic obstructive pulmonary disease (COPD) and asthma) (16). Unfortunately, these studies limited investigations to conditions whose pathophysiological relationships were clinically recognized. However, network-based analyses should hypothetically enable the investigation of inter-relationships between complex polygenic traits without *a priori* selection by integrating data across different levels of biological information (e.g., eQTLs, protein interactions, and GWAS traits). Network analyses that do not *a priori* restrict the tested conditions to those clinically recognized can further our ability to understand the comorbidity, development, and therapeutic avenues for pathologically related traits (14, 17, 18).

We hypothesized that a network analysis that integrates protein interaction data with lung and whole blood tissue-specific spatial gene regulatory networks (GRNs) would enable the identification of the complex traits and shared genetic risk associated with asthma.

## Materials and methods

### Data sources

- Gene expression levels (median transcripts per million; TPM) in whole blood were obtained from GTEx (https://gtexportal.org/home/datasets, GTEx_Analysis_2017-06-05_v8_RNASeQCv1.1.9_gene_median_tpm.gct.gz, 15/3/2021).
- All variants genotyped within whole blood samples were downloaded from GTEx (https://gtexportal.org/home/datasets, GTEx_Analysis_2017-06-05_v8_WholeGenomeSeq_838Indiv_Analysis_Freeze.lookup_table.txt, 4/01/2021).
- Single nucleotide polymorphisms (SNPs) and associated traits were downloaded from the GWAS Catalog (https://www.ebi.ac.uk/gwas/docs/file-downloads, 08/01/2022).
- Gene biotype information was obtained from GENCODE (http://ftp.ebi.ac.uk/pub/databases/gencode/Gencode_human/release_26/gencode.v26.annotation.gtf.gz, 05/06/2021).
- Gene-level constraint metrics were obtained from gnomAD (https://gnomad.broadinstitute.org/downloads#v2-constraint, gnomad.v2.1.1.lof_metrics.by_gene.txt, 11/01/2021).
- Gene start and end positions were obtained from GTEx (https://gtexportal.org/home/datasets, gencode.v26.GRCh38.genes.gtf, 17/02/2021).
- The DisGeNet (v7) API was used to obtain curated gene-disease associations. UMLS CUI to ICD10 disease mapping tables were downloaded from the DisGeNet repository (2/07/2022).
- ICD-10 to ICD-10-AM (11^th^ edition) mapping tables were downloaded from the Independent Hospital Pricing Authority (IHPA) (https://www.ihpa.gov.au/publications/icd-10-am-and-achi-mapping-tables, icd-10_2016_to_icd-10-am_eleventh_edition_0.zip, 7/8/2022)
- GRCh38.p13 was the human genome build used in this study.

### Construction of the lung and whole blood spatial gene regulatory networks

**Figure 1:**
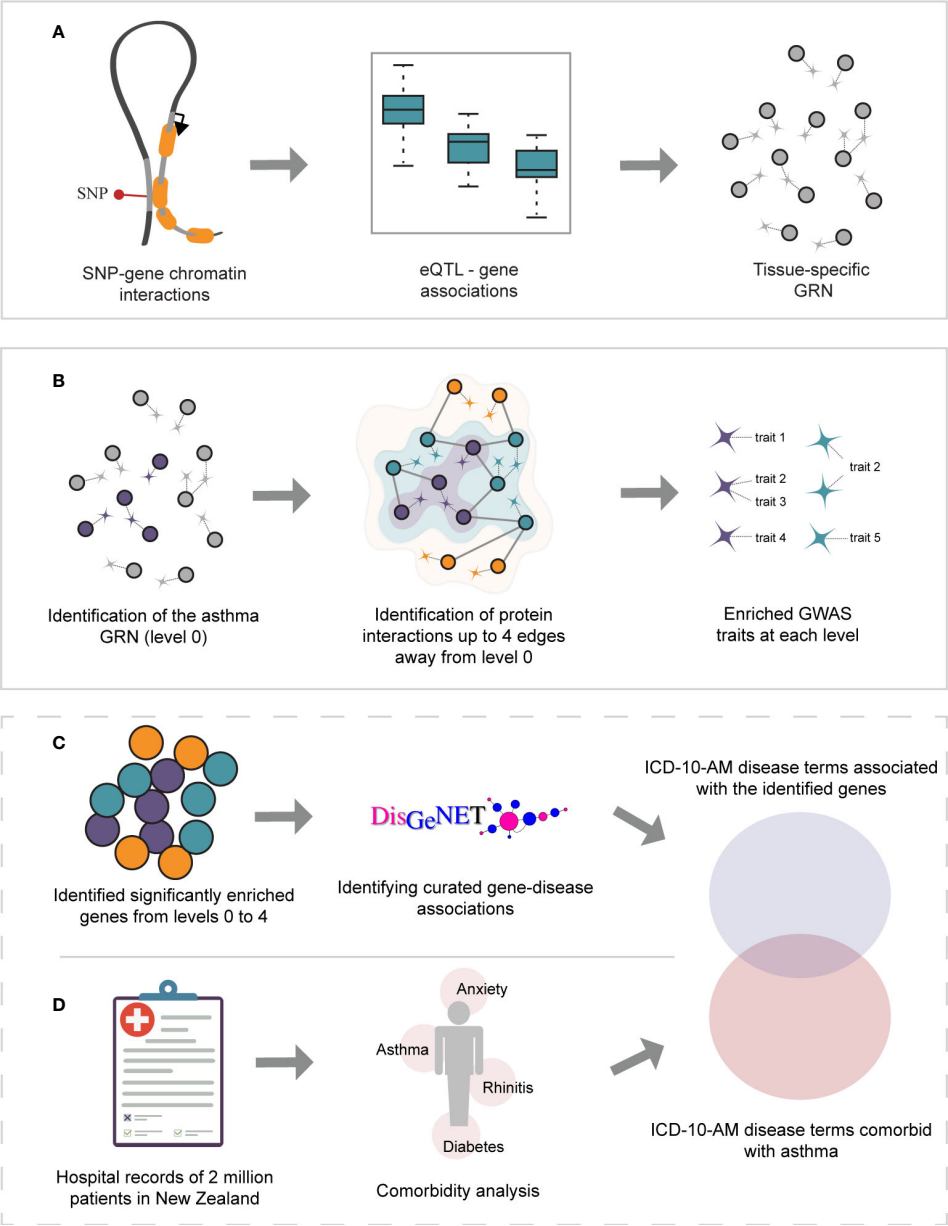
Study overview. **(A)** Gene regulatory networks were identified within GTEx lung and whole-blood tissues using spatially constrained eQTLs. **(B)** The asthma GRNs (level 0, depicted in purple) were identified within the lung and whole-blood GRNs. The asthma GRNs are comprised of asthma-associated spatial eQTLs, their target genes, and the remaining spatial eQTLs targeting them. The asthma GRNs were expanded (i.e., levels 1-4) by incorporating curated protein interactions (STRING and PROPER databases) that occur up to 4 edges away. For simplicity, only levels 1 and 2 are depicted (green and orange, respectively). The set of spatially constrained eQTLs associated with the genes at each level (i.e., 0-4) were queried against the GWAS catalog to identify traits enriched at each level (hypergeometric test, FDR ≤ 0.05, and 1000 sets of Monte Carlo simulation). **(C)** Gene-disease associations were identified for all genes using DisGeNet. UMLS CUI identifiers from DisGeNet were converted to ICD-10-AM disease terms. **(D)** A comorbidity analysis was performed on hospital records for New Zealand asthmatic patients.

The CoDeS3D pipeline (https://github.com/Genome3d/codes3d-v2) (19) was used to construct the tissue-specific GRNs. In brief, lung and blood primary cell line Hi-C data was downloaded and analyzed according to Rao et al. (20). The restriction enzymes used to prepare the libraries (i.e., HindIII and MboI) were used to digitally digest the GRCh38 genome into DNA fragments. SNPs (MAF ≥0.05) present within whole blood and lung samples (GTEx (11)) were used to interrogate the Hi-C libraries for interactions captured between a DNA fragment overlapping a gene (as defined by gencode v.26) and the DNA fragment containing the queried SNP (**Figure 1**). Interactions from adjacent fragments were ignored, and no maximum loop length was set for this analysis. Only SNP-gene interactions captured in more than one replicate for each Hi-C cell line/tissue were included in downstream analysis. Hi-C cell lines and GEO IDs of replicate samples can be found in **Supplementary Data 1**.

SNP-gene pairs were tested to identify eQTLs within each tissue [GTEx (11)]. The covariates adjusted for are sex, top 5 genotyping principal components, PEER factors, sequencing protocol, and sequencing platform. Finally, multiple testing correction (Benjamini-Hochberg) was performed, and spatial eQTLs with an adjusted p-value ≤0.05 were selected as significant. A SNP that: 1) physically contacts and 2) is associated with the transcription level of the interacting gene is termed a spatial eQTL (**Figure 1A**). Spatial cis-eQTLs associate with transcript levels of genes that are<1 Mb away from the spatial eQTL on the same chromosome while trans-intrachromosomal spatial eQTLs are associated with transcript levels of genes that are >1 Mb away on the same chromosome. Trans-interchromosomal spatial eQTLs are associated with transcript levels of genes that reside on different chromosomes.

### Identification of traits sharing molecular interactions with asthma

We constructed the multimorbid3D pipeline to identify traits associated or comorbid with an index condition. Scripts for the complete pipeline are accessible through https://github.com/Genome3d/multimorbid3D. All subsequent data analysis were performed using R version 4.0.1 (21) and RStudio version 1.3.959 (22). Briefly, asthma-associated SNPs (p = 1x10^-5^) were identified using a keyword search for the exact term “Asthma” in the GWAS Catalog (v1.0.2, 08/01/2022; **Supplementary Data 2**, (23)). The asthma-associated SNP set was then used to identify asthma-associated spatial eQTLs, and the genes they regulate within the lung GRN. These genes, and all the spatial eQTLs that regulate them (including spatial eQTLs associated with traits other than asthma), represent the base asthma GRN (or level 0).

Biologically similar and comorbid conditions often segregate in proximal protein interaction neighborhoods. Therefore, identifying proteins that interact with the asthma GRN may improve our understanding of the biology of asthma. As such, the asthma GRN was expanded to include curated protein interactions from STRING (24) (only the five highest-scoring interactions were used; of those, only interactions with a medium confidence score of ≥ 0.700 were considered; **Supplementary Data 3**, **4**; accessed 29/04/2022) and PROPER (25) that occur up to 4 edges away (**Supplementary Data 5**, **6**). For example, level 1 comprises the genes that encode the first neighbor proteins that share 1 edge (*i.e.*, directly interact) with level 0 proteins. Similarly, level 2 includes the genes for the second-neighbor proteins that share 1 edge with level 1 (and 2 edges with level 0) and so on. Spatial eQTLs that regulate the expression of the protein-coding genes on each level were identified by querying the lung GRN. The spatially constrained eQTLs associated with the protein-coding genes from levels 0 to 4 were queried against the GWAS Catalog to identify traits associated with these variants by GWAS. The hypergeometric distribution test was used to determine the enrichment of traits at each level:


(1) $$sf\left(Trait,\;Level\right)=\;1-\sum_{i=0}^{n}\frac{\left(\begin{array}{c} n\\ x_{i} \end{array}\right)\left(\begin{array}{c} M-n\\ N-x_{i} \end{array}\right)}{\left(\begin{array}{c} M\\ N \end{array}\right)}$$

Where N is the total number of spatial eQTLs identified at the given level, x is the total number of spatial eQTLs associated with a trait identified at the given level, M is the total number of unique SNPs in the GWAS Catalog, n is the total number of GWAS Catalog SNPs associated with the identified trait. Multiple testing correction was performed to identify significantly enriched traits (Bonferroni correction, FDR = 0.05).

To remove biases associated with the protein interaction network topology and to identify traits specific to asthma, this process was repeated 1000 times in a *Monte Carlo* simulation on a random *N* number of genes, where *N* is the size of the disease GRN (level 0) to be randomized. Traits, and their respective eQTL-gene pairs, having an adjusted p-value< 0.05 were selected as being significant (**Figure 1B**). Because there is no constraint on the SNP’s direction of effect, the identified traits could be positively or negatively associated with asthma.

This entire process was repeated using the whole blood GRN.

### Identifying curated gene-disease associations

Genes were assigned to diseases using DisGeNet (26). To reduce the number of false positives, we included only curated GDAs derived from CGI (27), PsyGeNET (28), ClinGen (29), CTD (human data) (30), UniProt (31), Orphanet (32), and the Genomics England PanelApp (33). Disease mappings were used to convert the UMLS CUI identifiers to ICD-10 disease terms. These were then converted to ICD-10-AM disease terms using the mapping tables provided by IHPA (accessed 07/08/2022) (**Figure 1C**).

### Comorbidity analysis

Patient diagnostic records (n = 2,051,661, 1,119,021 females and 2,051,658 males) from the 31^st^ of December 2015 to the 1^st^ of January 2021 were obtained from the New Zealand Integrated Data Infrastructure. Records of patients over the age of 100 were excluded from the analysis. From these records, conditions that are comorbid with asthma (ICD-10-AM 11^th^ edition code, J45.9) were identified using the comoRbidity R package (34). Odds ratio, relative risk, and comorbidity scores were calculated as follows:


(2) $$Odds\;ratio_{AB}=\;\frac{C_{AB}H}{C_{A}C_{B}}$$

(3) $$Relative\;risk_{AB}=\;\frac{C_{AB}N}{P_{A}P_{B}}$$

(4) $$Comorbidity\;score=\;log_{2}\left(\frac{observed\;C_{AB}\;+\;1}{expected\;C_{AB}\;+\;1}\right),\;expected\;C_{AB}=\;\frac{P_{A}P_{B}}{N}$$

Where A is asthma and B is the disease being tested, C*_A_* is the number of patients diagnosed with asthma, C*_B_* is the number of patients diagnosed with disease B, the number of patients diagnosed with both asthma and disease B is given by C*_AB,_* H denotes the number of patients without asthma and disease B, *N* is the total number of patients being tested. The prevalence of asthma and disease B is given by P*_A_* and P*_B_*, respectively. Diseases with less than 6 patients were excluded, as were diseases with a 95% confidence interval of odds ratio that overlaps 0 (**Figure 1D**). No clinical samples were accessed for this analysis.

### Ethics

The use of the Integrated Data Infrastructure within Stats NZ Data Lab was reviewed and approved by Statistics New Zealand (project number MAA2020-63) and the Auckland Health Research Ethics Committee (approval AH22495).

## Results

### Structures of the lung and whole blood-specific spatial gene regulatory networks

SNPs (n=40x10^6^, MAF≥ 0.05) within the GTEx dataset were downloaded (dbGaP accession: phs000424.v8.p2; approved project number: #22937) and screened for spatial eQTLs in lung and whole blood (**Figure 1**) using the CoDeS3D algorithm (19). The resulting lung GRN comprises 873,133 spatial eQTL-gene interactions (740,028 spatial eQTLs regulating 15,855 genes expressed in the lung) and has more cis-acting (eQTL-gene are separated by<1Mb) than trans-intrachromosomal (eQTL-gene pair are separated by ≥1Mb on the same chromosome) or trans-interchromosomal spatial eQTLs (eQTL-gene pair are located on different chromosomes). The lung GRN has been previously described in more detail (35). The whole blood GRN comprises 1,713,885 spatial eQTLs-gene interactions (1,077,379 spatial eQTLs regulating 14,871 genes expressed in whole blood). The blood GRN also has more cis-acting (n=1,634,655) than trans-intrachromosomal (n=67,014) or trans-interchromosomal spatial eQTLs (n=12,216) (**Figures S1, S2**). A detailed description of the blood GRN can be found in **Supplementary Note 1**, **Figures S3**, **S4**.

### SNPs from 68 traits are associated with asthma through pleiotropic genes

**Figure 2:**
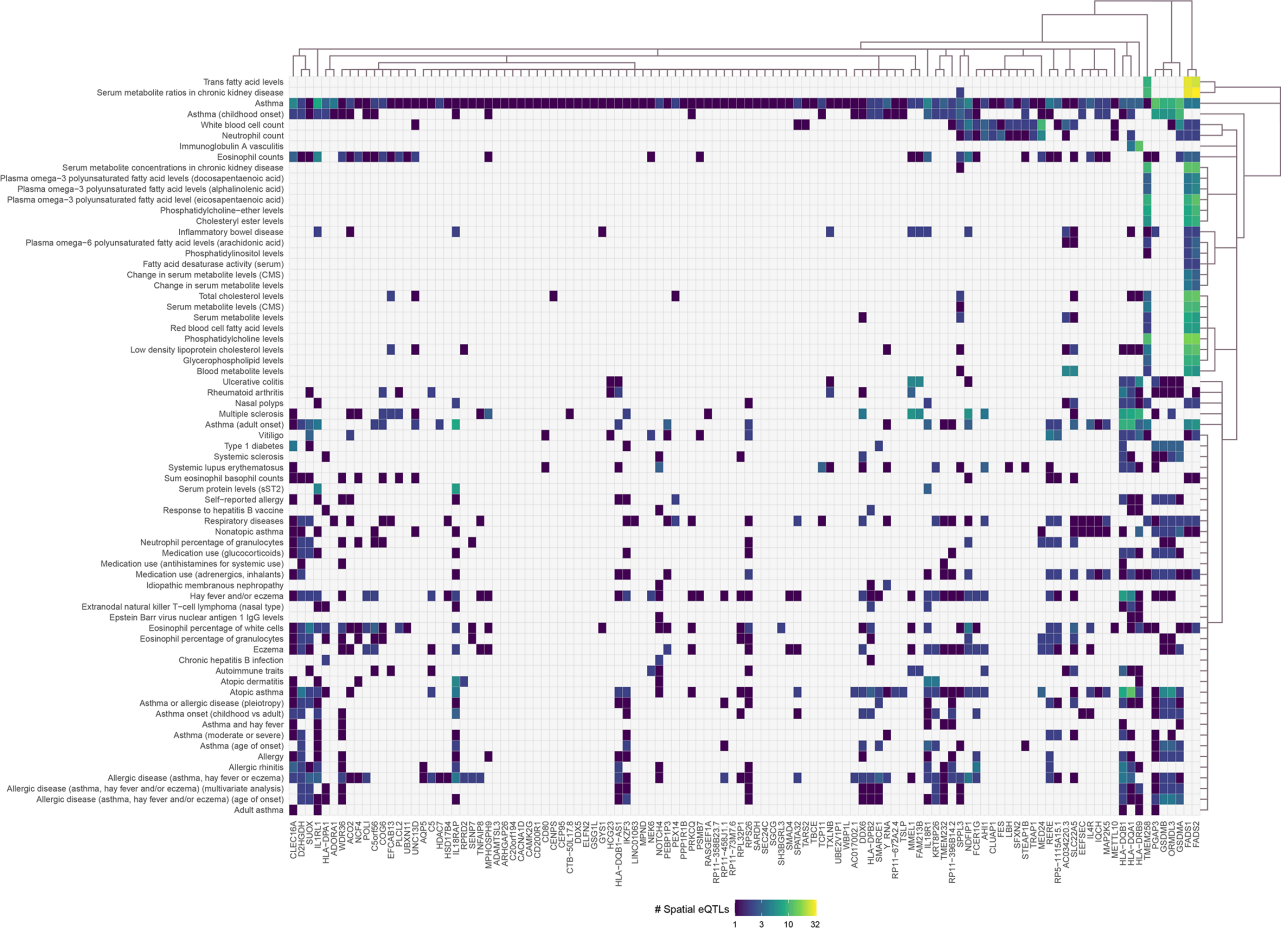
Asthma-associated spatial eQTLs regulate pleiotropic genes within the asthma lung-specific GRN. Convex biclustering of trait-eQTL-gene interactions identified pleiotropic genes at level 0 within the asthma lung-specific GRN. For each eQTL, the x-axis represents the target gene, and the y-axis represents the associated trait. Only eQTL-gene pairs associated with traits that passed the Monte Carlo simulation are included.

Asthma SNPs (n=383; **Supplementary Data 2**) were mapped onto the lung GRN (**Figure 1**) to identify 112 genes (i.e., the asthma L-GRN) whose transcript levels are regulated by spatial eQTLs associated with asthma (n=155 [153 cis interactions and 2 trans-intrachromosomal interactions], 40.5% of the asthma-associated SNPs) and spatial eQTLs associated with an additional 68 traits (n=480, **Figure 2**). Running the asthma-associated SNPs through FUMA’s SNP2GENE function prioritized 104 of the 112 genes identified using our pipeline (36) (see **Supplementary Note 2**). The asthma L-GRN includes HLA genes and other genes previously reported to be associated with asthma (e.g., *PRKCQ* (37), *GSDMA* (38), and *GSDMB* (39)). Of the 68 traits that were identified as associating with asthma through pleiotropic genes, 9 are endotypes of asthma (e.g., childhood-onset, adult-onset, nonatopic, atopic, moderate or severe asthma), 9 represent allergic disease (e.g., allergic rhinitis, allergic disease [asthma, hay fever or eczema]), 7 represent blood cell counts (e.g. eosinophil counts, neutrophil count), 12 are autoimmune diseases (e.g., rheumatoid arthritis, type 1 diabetes, vitiligo, systemic lupus erythematosus [SLE], inflammatory bowel disease), 23 pertain to metabolite levels (e.g., red blood cell fatty acid levels, omega-6 polyunsaturated fatty acid levels), 3 are related to drugs used for asthma treatment and to relief allergy symptoms (e.g., medication use: adrenergics, inhalants; glucocorticoids; and antihistamines for systemic use) and 5 “other” traits (i.e., nasal polyps, idiopathic membranous nephropathy, extranodal natural killer T-cell lymphoma nasal type (ENKTL-N), respiratory disease, response to hepatitis B vaccine). The identified pleiotropic genes serve as connectors between asthma and the identified traits (**Figure 2**, **Supplementary Data 7**).

The same process was repeated using the blood GRN to identify 188 pleiotropic genes (i.e., the asthma B-GRN) regulated by spatial eQTLs associated with asthma (n=57 [56 cis and 1 trans-intrachromosomal interaction], 14.9% of asthma-associated SNPs) and 70 other traits (n=815). A hypothesis test for one population proportion (one-sample proportion test) identified that the proportion of asthma-associated spatial eQTLs in the lung GRN is significantly higher than in the blood GRN (p< 0.00001), consistent with the central role of the lungs in asthma. The enriched traits within the asthma B-GRN were comparable to those identified for the asthma L-GRN, except for some psychological traits (e.g., depressive symptoms, schizophrenia (MTAG), and feeling worried) and cancers (e.g., cervical cancer, follicular lymphoma, **Figure S5**).

### Protein interaction networks reveal 12 traits that interact with asthma

**Figure 3:**
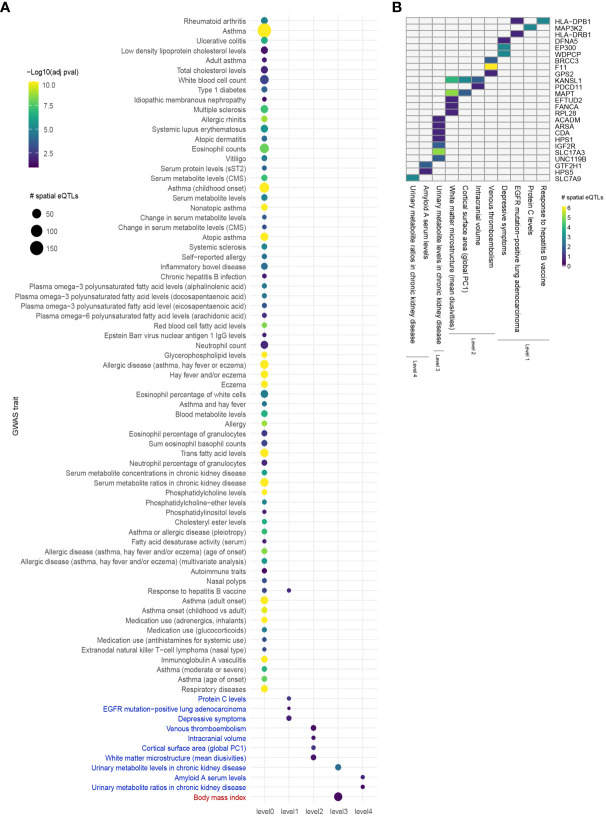
Integrating an expanded protein-protein interaction network with the lung GRN identifies conditions associated with asthma. **(A)** GWAS traits enriched for spatial eQTLs at levels 0 to 4 within the extended asthma L-GRN; circle size indicates the number of spatial eQTLs, and circle color indicates the statistical significance of enrichment. Traits in black are associated with spatial eQTLs that regulate the expression of level 0 genes. Traits in blue or red are associated with spatial eQTLs that regulate protein-coding genes having curated interactions in STRING or PROPER, respectively. **(B)** Trait-spatial eQTL-gene interactions occurring at levels 1 to 4 of the asthma L-GRN. For a given spatial eQTL or a set of spatial eQTLs, the x-axis represents the target gene (only genes with interactions in STRING are shown), and the y-axis represents the associated trait. Only spatial eQTLs-gene pairs associated with traits that passed the Monte Carlo simulation are included.

Comorbid and biologically similar diseases tend to segregate in modules within the same network neighborhood (20, 23). Therefore, we translated the lung GRN into a protein-protein interaction network (PPIN) to include curated protein interactions occurring in STRING (medium confidence score ≥ 0.700) and PROPER. Briefly, starting from the 112 genes that are regulated by asthma-associated spatial eQTLs, the asthma GRN was expanded using curated protein interactions to form 4 concentric PPIN levels (**Figure 1B**). The 4 PPIN levels specific to the lung contained a total of 1,478 proteins before bootstrapping, at which point the network was reduced to a set of 37 protein-coding genes. The spatial eQTLs regulating the genes identified at each level were then identified from the lung GRN. 6 interacting proteins were identified at level 1 (1 edge from level 0), which were regulated by 15 spatial eQTLs within the lung GRN. At level 2, 24 spatial eQTLs targeted transcript levels of 9 genes. At level 3, 51 spatial eQTLs targeted transcript levels of 20 genes. Finally, at level 4, 6 spatial eQTLs targeted transcript levels of 3 genes. The detected spatial eQTLs were enriched within 4 GWAS traits at level 1 (hypergeometric test; FDR ≤ 0.05 and 1000 sets of Monte Carlo simulation), 4 traits at level 2, 2 traits at level 3, and 2 traits at level 4 (**Figure 3**, **Supplementary Data 7**).

**Figure 4:**
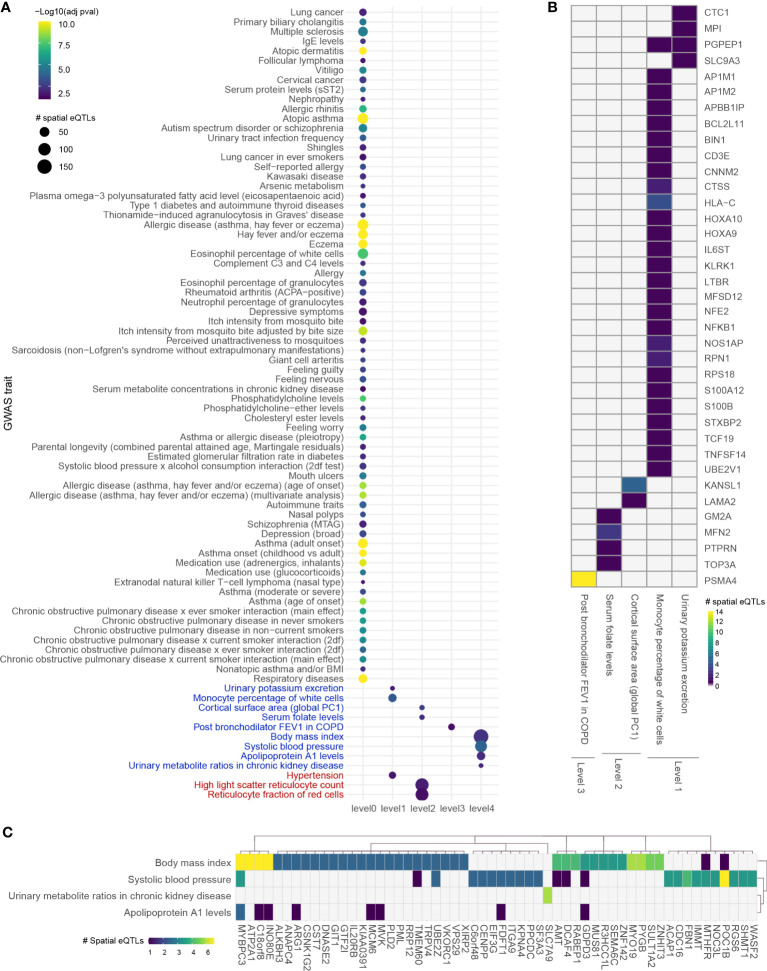
Integrating an expanded protein-protein interaction network with the blood GRN identifies conditions associated with asthma. **(A)** GWAS traits enriched in levels 0 to 4 within the extended asthma B-GRN; circle size indicates the number of spatial eQTLs, and circle color indicates the statistical significance of enrichment. Traits in black are associated with spatial eQTLs that regulate the expression of level 0 genes. Traits in blue or red are associated with spatial eQTLs that regulate protein-coding genes having curated interactions in STRING or PROPER, respectively. **(B)** Heatmap showing trait-spatial eQTL-gene interactions occurring within levels 1 to 3 of the extended asthma B-GRN (only genes with interactions in STRING are shown). **(C)** Trait-spatial eQTL-gene interactions occurring at level 4 of the extended asthma B-GRN. For a given spatial eQTL in B or C, the x-axis represents the target gene, and the y-axis represents the associated trait. Only spatial eQTLs-gene pairs associated with traits that passed the Monte Carlo simulation are included. Of those, only genes regulated by ≥ 2 spatial eQTLs are shown in **(C)**.

Expanding four levels away from asthma B-GRN (level 0) identified 2,471 proteins over 4 levels before bootstrapping, at which point it reduced to a set of 325 protein-coding genes within the blood GRN. Of those, 35 genes were regulated by 52 spatial eQTLs at level 1, 184 spatial eQTLs regulated 156 genes at level 2, 1 gene was regulated by 14 spatial eQTLs at level 3, and 276 spatial eQTLs regulated 146 genes at level 4. Analysis for enrichment in GWAS traits identified 4 enriched traits at levels 1, 2, and 4 and 1 trait at level 3 (**Figure 4**).

### Gene-disease association analysis identifies asthma comorbidities replicated in New Zealand asthma patient health records

Complex diseases such as rhinitis, metabolic syndrome, and dermatitis have been identified as comorbidities in asthma patients, suggesting that shared molecular pathways contribute to their development (40). Our analysis identified 126 GWAS traits interacting with asthma (**Supplementary Data 7**, **Figures 3**, **4**). However, while these traits provide insights into asthma pathology, many do not represent disease conditions (e.g., amyloid A serum levels). Therefore, we queried DisGeNet with the genes from each level (levels 0-4) to identify their associated diseases. 35 genes were associated with 306 curated UMLS CUI disease identifiers (**Supplementary Data 8**) that map to 57 ICD-10 disease terms and 53 ICD-10-AM disease terms.

**Figure 5:**
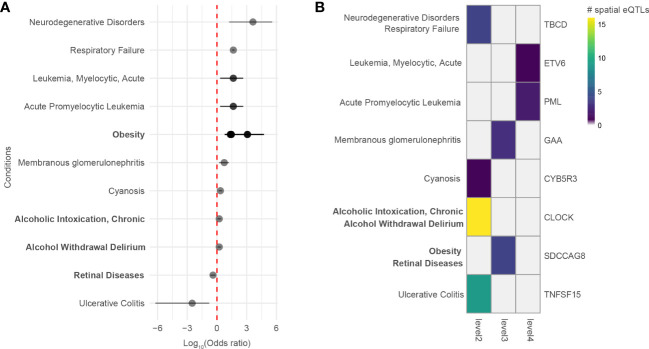
Genes 2 to 4 levels away from the asthma B-GRN and L-GRNs are associated with comorbidities observed within hospitalized patient records. **(A)** Diseases that are positively or negatively associated with asthma in patients hospitalized in New Zealand between 31/12/2015 and 01/01/2021. Diseases were identified using the ICD-10-AM disease terms. Multiple odds ratios exist for some diseases with more than one ICD-10-AM disease term or UMLS CUI identifier. For example, acute promyelocytic leukemia (UMLS CUI = C0023487, ICD-10-AM = C92) maps to acute promyelocytic leukemia [PML], in remission (ICD-10-AM C9241) and acute promyelocytic leukemia [PML], without mention of remission (ICD-10-AM C9240). Asthma B-GRN, normal font; asthma L-GRN, bold font. **(B)** Disease terms from A that have reported associations with genes located 2 to 4 levels away from the asthma B-GRN (normal font) and asthma L-GRN (bold font).

A comorbidity analysis was performed using hospitalization records in New Zealand to identify which disorders co-occur in asthma patients. Of 2,051,661 hospitalized patients, 26,781 were diagnosed with asthma, equating to an asthma disease prevalence of 1.305% among hospitalized patients in New Zealand between 31/12/2015 and 01/01/2021. 194 ICD-10-AM terms were statistically associated with asthma (q-value<0.05). These include conditions that are not necessarily disorders, e.g., complications following a procedure, drug use, etc. Of the disorders, the most significant comorbidities of asthma (q-value<0.001, ranked by odd ratio) included family history of asthma and other chronic lower respiratory diseases, polyarteritis with lung involvement [Churg-Strauss], drug-induced Cushing’s syndrome, unspecified acute lower respiratory infection, unspecified dermatitis, occupational exposure to toxic agents in other industries and bronchiectasis. A full list of identified disease terms associated with asthma can be found in **Supplementary Data 9**. Of the 194 ICD-10-AM disease terms, 9 terms (mapping to 11 UMLS CUI disease terms) were also identified in the gene-disease association analysis (p-value = 0.0051, Fisher’s exact test). All identified diseases had increased odds of being comorbid with asthma except for retinal diseases and ulcerative colitis (**Figure 5A**). The identified diseases were associated with 8 genes (**Figure 5B**) that fall 2 to 4 levels away from the asthma L-GRN and B-GRN.

### 142 genes modulating asthma-trait interaction have known drug-gene interactions

We compared the genes identified within the extended asthma L-GRN and B-GRNs (hypergeometric test, FDR ≤ 0.05 and 1000 sets of Monte Carlo simulation) with genes from a) the druggable genome (41) and b) the drug-gene interaction database [DGIdb (42)] to identify opportunities for drug repurposing. Of the 606 genes we identified, 139 were present in the druggable genome, and 142 have known drug interactions (**Supplementary Data 10**).

Valette et al. used Mendelian Randomization to prioritize 50 genes regulated by non-spatial cis eQTLs in the blood as being causally associated with asthma (43). Comparison with the asthma L-GRN and B-GRNs identified that 22/50 of those genes were pleiotropic genes at level 0 in our analysis (**Supplementary Data 10**). Of those, 12 genes were regulated by the same eQTL in both studies, namely *UBAC2*, *TEF*, *TDRKH*, *SUOX*, *SLC22A5*, *SCRN2*, *RPS26*, *MEI1*, *IKZF3*, *GSDMB*, *FADS1*, and *CSDC2*.

## Discussion

The molecular and biological mechanisms that influence complex phenotypes interact across multiple levels of biological information. In this study, we used lung and whole blood-specific spatial GRNs to identify conditions that interact with asthma. The GRNs connected asthma-associated spatial eQTLs with spatial eQTLs associated with other traits by integrating information on curated protein-protein interactions, thereby identifying various traits interacting with asthma, many of which had previously reported associations with asthma. It additionally identifies the genetic variants and proteins bridging these traits to asthma.

This study is not without limitations. Firstly, asthma-trait associations can only be identified using variant-gene pairs that physically interact and will only involve traits previously investigated by GWAS. Additionally, not all genetic variants can be queried due to a bias for participants of primarily European ancestry within GWAS studies (44). Secondly, our method relies on regulatory connections affecting genes that have known interactions in STRING and PROPER, where STRING integrates various sources as evidence for protein interaction (e.g., co-expression, text-mining, and genomics context prediction channels) and PROPER, which at its current state limits interactions to those identified in 3 cell lines. Moreover, spatial eQTLs whose target genes do not form protein-protein interactions, or those forming unknown interactions, will be missed by our analysis. The third limitation is that the lung and blood GRNs we constructed are not dynamic (i.e., each represents a snapshot of captured regulatory and protein interactions) and are thus subject to change. Fourthly, our analysis combines information across multiple levels of biological organization that do not originate from the same biological samples [i.e., eQTL data from GTEx (45), Hi-C datasets [**Supplementary Data 1**], population studies [GWAS (23)], and protein-protein interaction data from curated and experimental datasets (24, 25)]. Moreover, while a disease SNP and a spatial eQTL may not necessarily overlap, they could colocalize within the same linkage disequilibrium (LD) block. These LD effects have not been explored in this particular analysis. Finally, disease risk is associated with both rare and common genetic variation. Therefore, the focus on common variation overlooks disease risk associated with the convergence of common and rare effects. Notwithstanding these limitations, the approach we have outlined improves our ability to identify how common genetic variation connects traits that interact with asthma through described molecular pathways.

Asthma is a complex immune-mediated inflammatory airway disease whose features include airway remodeling and respiratory obstruction (46, 47). We identified that the genes regulated by asthma-associated spatial eQTLs in the lung are also regulated by spatial eQTLs associated with various asthma endotypes, allergic and autoimmune disorders, metabolite levels, and traits related to the use of asthma medication. For example, variants associated with SLE risk, a known comorbidity of asthma (pooled odds ratio (OR): 1.37; 95% CI 1.14–1.65; I2 = 67%) (48), regulate pleiotropic genes that are regulated by asthma-associated spatial eQTLs. The identification of 22 genes causally related to asthma through Mendelian Randomization as being pleiotropic and linked to other traits in our analysis further supports the existence of shared molecular pathways that could contribute to comorbidity risk (43). It also raises the spectre that those not identified in our network either act in another tissue or are not transcriptionally active at the developmental stages at which data for these tissues were obtained.

Metabolite-associated traits (n=23 traits) were the single most enriched category of traits identified as sharing genetic and protein mechanisms with asthma. This included omega-3 and omega-6 polyunsaturated fatty acid levels (e.g., arachidonic acid), which have been associated with the attenuation and upregulation of asthmatic pathways (49). The metabolism of arachidonic acid can produce cysteinyl leukotrienes, which is overexpressed in asthma patients and has recently been linked to the production of IL-4, IL-5, and IL-13 by binding ILC2 and Th2 cells (50). Our analysis identifies 5 arachidonic acid-associated spatial eQTLs and 5 target genes (i.e., *FADS1*, *FADS2*, *TMEM258*, *SLC22A5*, and *AC034220.3*) that potentially affect this interaction. Notably, decreased activity of *FADS1* and *FADS2* (fatty acid desaturase 1 and 2) are markers of asthma progression (51).

Proteins do not work independently of each other. Therefore, we analyzed protein-coding genes that directly and indirectly interact with protein-coding genes on the asthma GRNs (level 0). This identified traits that directly interact with asthma (level 1 traits) and traits that indirectly interact with asthma (levels 2 to 4). Within the lung GRN, all the enriched traits, apart from ENKTL-N, have reported associations with asthma. These include venous thromboembolism (hazard ratio (HR): 3.24, (52)), amyloid A serum levels (53), protein C levels (54), intracranial volume (55), white matter microstructure (mean diusivities) (56, 57) and depressive symptoms (58). Notably, of the 80 traits enriched across levels 0-4, only 8 were identified when running this analysis using COPD-associated SNPs, (i.e., white blood cell count, vitiligo, serum metabolite levels, atopic asthma, asthma (adult onset), cortical surface area [global PC1], urinary metabolite levels in chronic kidney disease and urinary metabolite ratios in chronic kidney disease), despite the use of the same lung GRN, suggesting disease-specific results (35). Of the 82 traits identified in the blood GRN, all had known associations with asthma, apart from 8 traits whose association with asthma is yet to be confirmed, i.e., primary biliary cholangitis, nephropathy, giant cell arteritis, parental longevity, systolic blood pressure x alcohol consumption interaction, ENKTL-N, high light scatter reticulocyte count and reticulocyte fraction of red cells traits. As for the known asthma-associated traits identified using the asthma B-GRN, they include serum folate levels [OR=1.45, 95% CI 1.05–2.02, (59)], urinary potassium excretion (60, 61), monocyte percentage of white cells (62, 63), and body mass index [relative risk: 1.21, 95% CI 1.16–1.26, (64)]. We propose that studies investigate the novel associations identified in our study; traits such as high light scatter reticulocyte count and reticulocyte fraction of red cells are of particular importance since they could serve as potential biomarkers of asthma.

Despite reported links between asthma and an increased risk for lung cancer [HR 1.29; 95% CI 0.95–1.75 (65)], the mechanism remains unknown. Chronic inflammation (66), structural and functional changes of the lungs [e.g., thickening of the bronchial wall, fibrosis, and formation of scar tissue (67, 68)] could contribute to the asthma-lung cancer association. However, our analysis implicates 57 spatial eQTLs targeting 17 genes in the asthma-lung cancer interaction. Increased expression of *BTN3A2*, one of these genes, in resting T cells is causally associated with a lower risk of asthma development (69). Notably, increased expression of *BTN3A2* is associated with a favorable prognosis in lung cancer (70).

Our approach identified traits that are associated with asthma. However, many of these traits are not recognized diseases. Thus, we used DisGeNet to identify curated disease associations for the genes within the PPINs. Of the diseases we identified as associated with these genes, 9 were identified as comorbid with asthma in New Zealand patients hospitalized with asthma and 2 occurred at higher levels within the non-asthmatic hospitalized population (i.e., retinal diseases and ulcerative colitis). This is consistent with a previous observation that there is no increased risk of ulcerative colitis in patients diagnosed with asthma (71) but inconsistent with others (71, 72). Notably, ulcerative colitis was associated with the pleiotropic *TNFSF15* gene (within the asthma L-GRN), which is associated with both childhood asthma (73) and ulcerative colitis (74).

The strength of the network approach we developed is the provision of molecular insights for the identified asthma-trait interactions. However, questions about the significance of finding risk variant-gene associations with blood (e.g., white blood cell count) and brain (e.g., intracranial volume, white matter microstructure) and neurological (e.g., depressive symptoms) related traits using a lung-specific GRN remain. It should be noted that while the GRNs are tissue-specific, most GWAS studies are carried out using blood samples. Traits are thus identified if their associated variant is: 1) GWAS significant; and 2) a spatial eQTL in the tissue of interest. This may help explain why some blood and brain-related disorders were observed despite using a lung-specific GRN. It may also reflect interconnectivity between whole-body organ systems, a study bias concerning gene and protein function, or the conserved action of regulatory sites (marked by spatial eQTLs) across tissue types. Further work will be required to determine which of these possible explanations is or are relevant.

Our *de novo* discovery approach can identify conditions comorbid with asthma without *a priori* selection of the interacting phenotypes. In so doing, it prioritizes phenotypes for which genetic variation and biological mechanisms interact and potentially affect disease presentation. The molecular connections we have identified in this study represent high-value targets for subsequent investigation into asthma development, comorbidity, and future therapeutic development.

## Data availability statement

The datasets presented in this study can be found in online repositories. The names of the repository/repositories and accession number(s) can be found in the article/**Supplementary Material**. Scripts used for data analysis are available at https://github.com/Genome3d/asthma_multimorbidities.

## Ethics statement

The studies involving human participants were reviewed and approved by Auckland Health Research Ethics Committee and Statistics New Zealand. Written informed consent for participation was not required for this study in accordance with the national legislation and the institutional requirements.

## Author contributions

RZ conceptualized, performed analysis, interpreted data, and wrote the manuscript. TF contributed to conceptualization, data analysis, data interpretation and manuscript revision. JO’S directed the study, contributed to data interpretation and data analysis, and co-wrote the manuscript. All authors read and approved the final manuscript.
