# Supplementary material for: De novo identification of complex traits associated with asthma
Source: Front Immunol. 2023 Aug 23;14:1231492. doi: 10.3389/fimmu.2023.1231492 (PMC10480836; doi:10.3389/fimmu.2023.1231492)
Supplement: Supplementary file 11 [file DataSheet_11.docx]

Supplementary Material

# Supplementary Data

Supplementary Data 1 – 10 are available at frontiers online.

The lung spatial gene regulatory network: [10.6084/m9.figshare.20205644.v1](https://doi.org/10.6084/m9.figshare.20205644.v1)

The whole blood spatial gene regulatory network: [10.17608/k6.auckland.17067953.v1](https://doi.org/10.17608/k6.auckland.17067953.v1)

The left ventricle spatial gene regulatory network: [10.17608/k6.auckland.18593432.v1](https://doi.org/10.17608/k6.auckland.18593432.v1)

The adult brain cortex spatial gene regulatory network: [10.17608/k6.auckland.18592811.v1](https://doi.org/10.17608/k6.auckland.18592811.v1)

# Supplementary Notes

**Supplementary Note 1** – Structure of the blood GRN

Genes regulated in trans in the blood GRN were enriched for developmentally essential genes (Supplementary Fig 2). Correlating the number of whole blood spatial eQTLs against the number of GTEx SNPs per chromosome identifies chromosomes 4, 8, 9, 13, and 18 as having fewer spatial eQTLs than expected and chromosomes 6, 17, and 19 as having more spatial eQTLs than expected, with chromosome 6 having the greatest enrichment (Supplementary Fig. 2d and 3). While the peak on chromosome 6 could represent a study bias associated with the HLA locus, it is possible that the peak in ratio of spatial eQTL-gene interactions/SNP reflects a regulatory feature of the HLA region, consistent with the observation of additional tissue-specific spikes on chromosomes 7, 18, and 19 (Supplementary Fig. 4).

**Supplementary Note 2** - Functional mapping and annotation in the lung using FUMA

To compare the asthma L-GRN genes (level 0 genes) identified by our pipeline to those prioritized by FUMA, we ran the asthma-associated GWAS SNPs through FUMA’s SNP2GENE function. The SNPs were mapped to 1) lung eQTLs (eQTL mapping) and 2) chromatin contact interactions (chromatin interaction mapping). GTEx v8 lung data was used to perform the lung cis-eQTL mapping (nominal pvalue <0.05). Chromatin interaction mapping was performed using Hi-C data of lung tissue (GEO accession number GSE87112, pvalue ≤0.05). This prioritized 313 genes by FUMA, while our pipeline prioritized 112 genes (level 0 genes) after bootstrapping. The lower number of genes identified by our pipeline can be attributed to the additional filtering applied in the identification of level 0, i.e., hypergeometric testing followed by multiple testing correction and bootstrapping (Monte Carlo simulation). Of the 112 genes identified by our pipeline, 104 were also identified by FUMA, and 8 genes were unique to our pipeline. Of the 104 shared genes, 67 were identified by both pipelines as being spatially regulated (i.e., eQTL physically contacts the gene as identified by chromatin interaction mapping), and 37 genes were identified by our pipeline as spatially regulated and by FUMA as regulated only by non-spatial eQTLs (i.e., no evidence for chromatin interaction was identified by FUMA). The differences in the chromatin interaction results could be attributed to the different processing of the Hi-C data, where FUMA uses Fit-Hi-C to compute the significant connections while our pipeline analyses them as per Rao et al. (please see methods). Moreover, we consider cis, trans-interchromosomal, and trans-intrachromosomal eQTLs in our pipeline, which we calculate using TensorFlow as part of the CoDeS3D pipeline (methods); whereas FUMA uses only cis eQTLs downloaded from GTEx. Finally, both pipelines use FDR correction. Thus, rank scores will differ between pipelines.

# Supplementary Figures


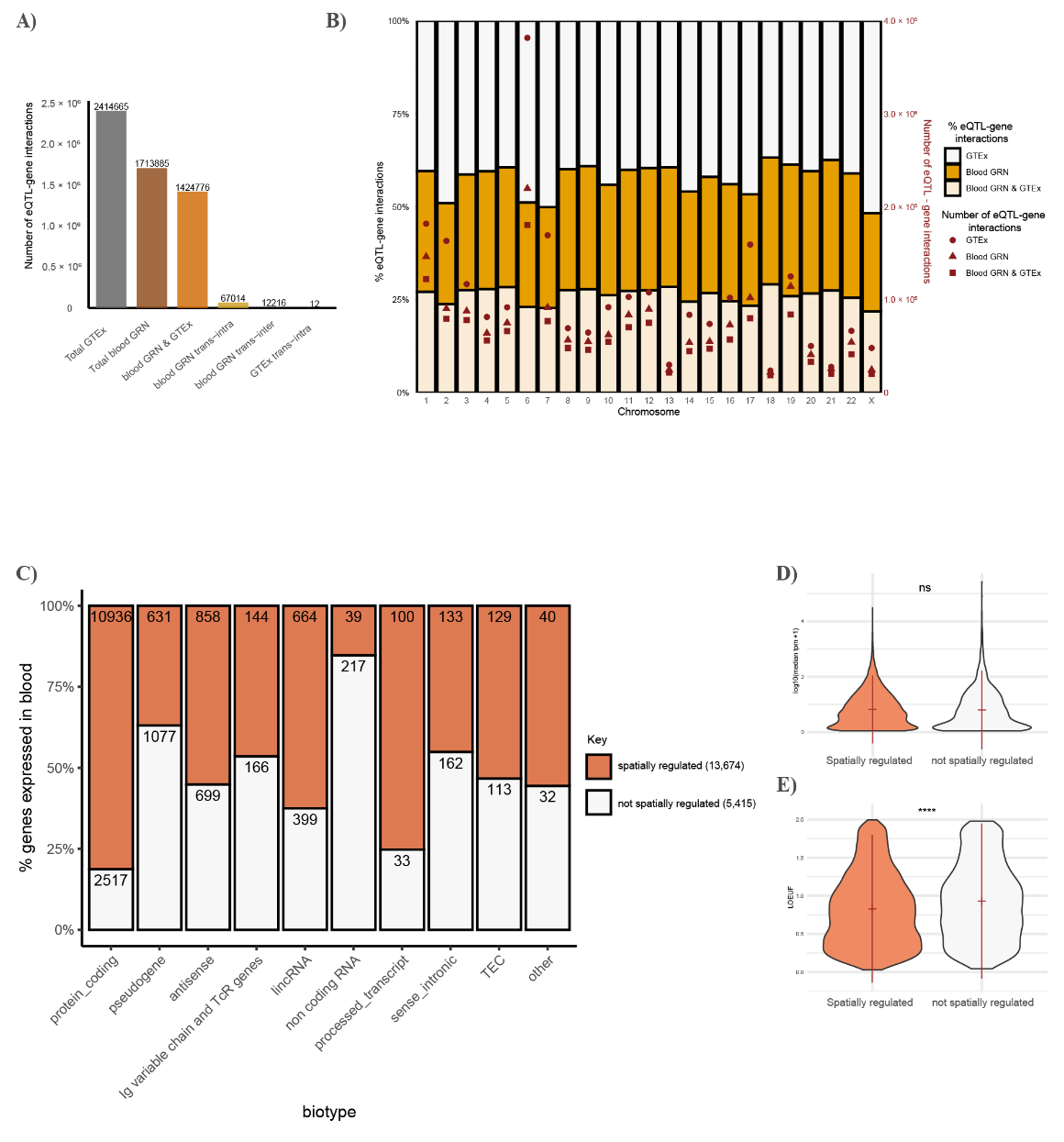


**Supplementary Figure 1. eQTL-targeted protein-coding genes are significantly more intolerant to loss of function than genes that are not targeted by eQTLs. A)** Number of eQTL-gene interactions in GTEx v8 compared to spatial eQTL-gene interactions observed in whole blood, classified by interaction type. **B)** Proportion of eQTL-gene interactions across chromosomes. **C)** Proportion of spatially regulated and non-spatially regulated genes expressed in whole blood (median TPM >0.1) classified by biotype. **D)** expression level and **E)** tolerance to loss of function of protein-coding genes in whole blood. In both D and E, the mean is shown in red. NS, not significant, ****P <1 × 10−4 (t-test).


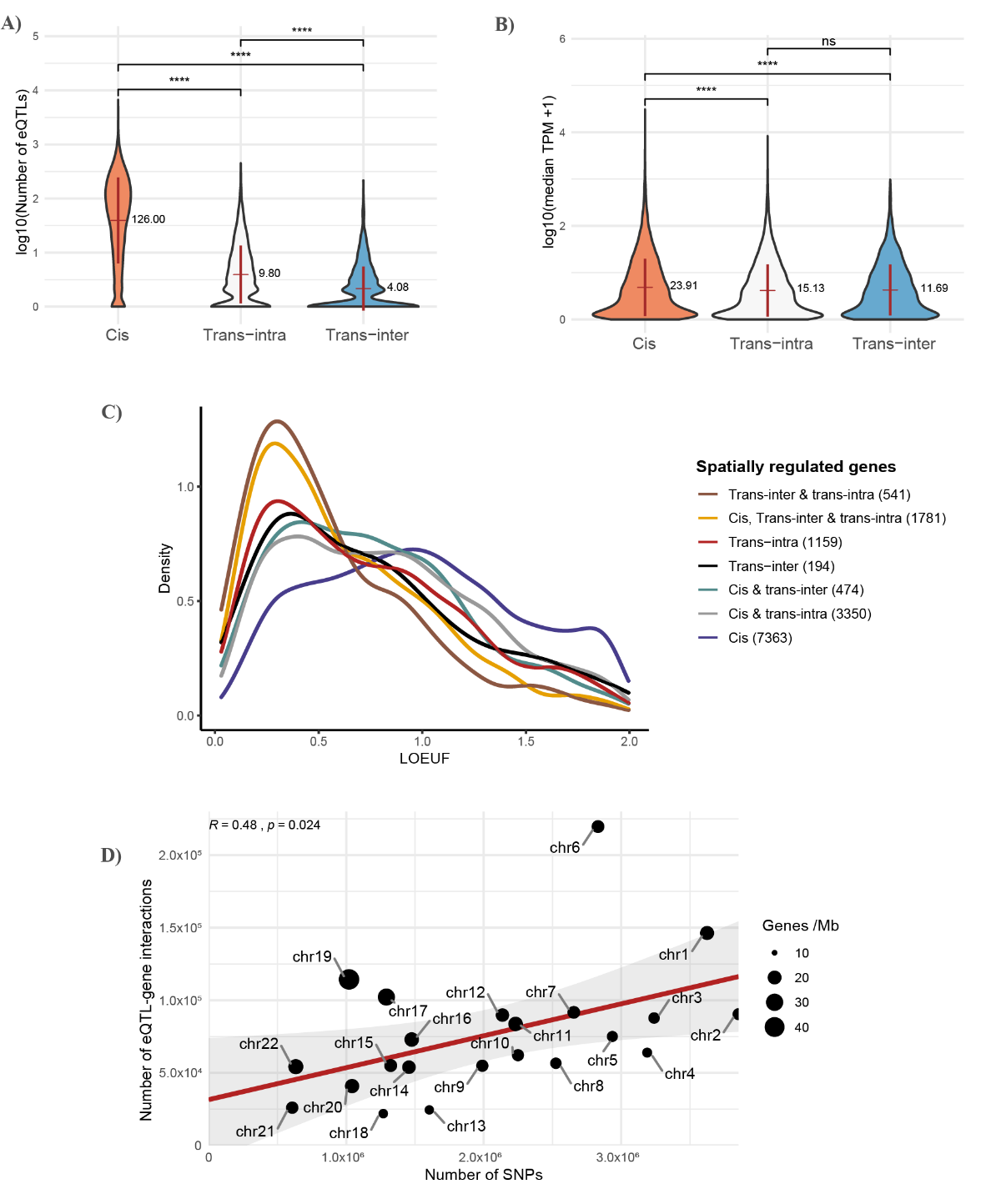


**Supplementary Figure 2. Characteristics of the blood GRN.** Mean (red line) and standard deviation of **A)** the number of spatial eQTLs per gene grouped by interaction type and **B)** gene expression grouped by interaction type. In both A and B, the mean of the untransformed data points is also denoted. **C)** Tolerance to loss of function of spatially regulated genes grouped by interaction type. **D)** Correlation of blood GRN eQTLs and all variants genotyped from whole blood samples obtained from GTEx v8. NS, not significant, ****P <1 × 10−4 (t-test).


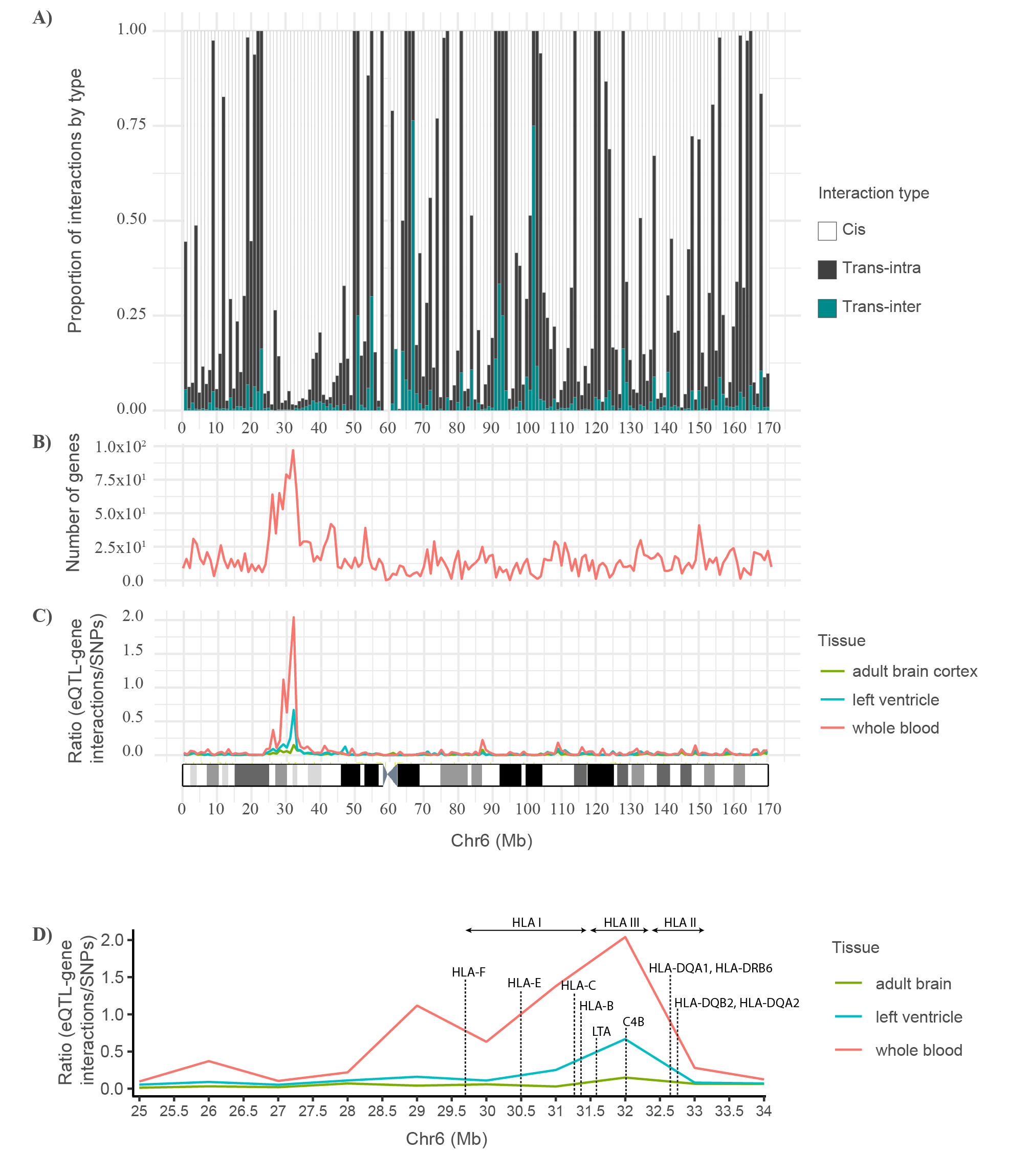


**Supplementary Figure 3. eQTL-gene interactions spike within the HLA region of chromosome 6 in whole blood but not in adult brain. A)** Proportion of spatial eQTL-gene interactions across chromosome 6 grouped by type **B)** Gene density across chromosome 6. **C)** Ratio of blood GRN eQTL-gene interaction to SNPs genotyped in chromosome 6; G-banding ideogram of chromosome 6 is shown below. **D)** magnified view of B showing the position of eQTL targeted genes in the HLA region. A sliding window of 100 Mb was used for all plots. The adult brain and left ventricle GRNs were constructed using the same method as the blood and lung GRNs (see data availability).

­­
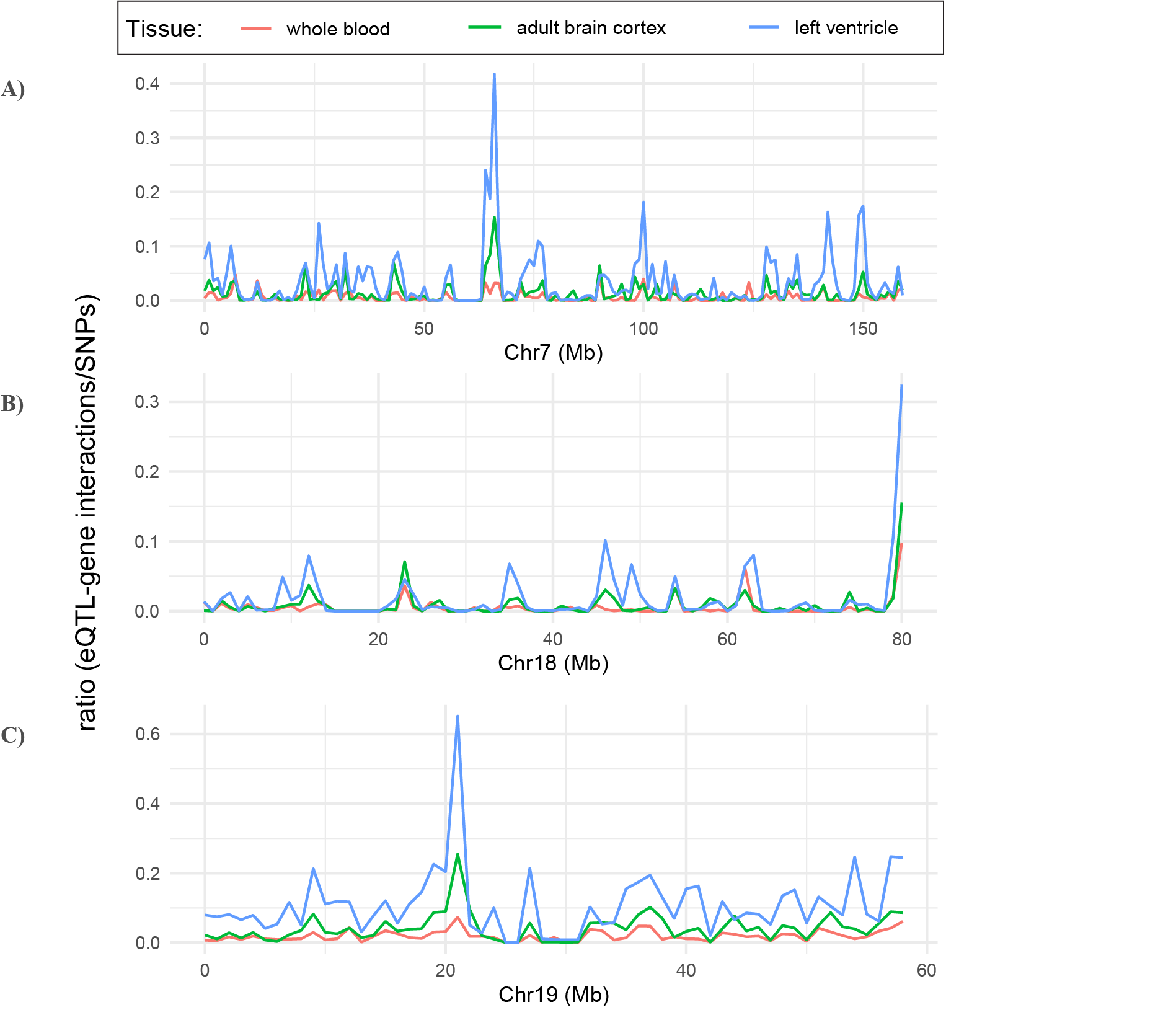
­­

**Supplementary Figure 4. Tissue-specific spikes in the ratio of eQTL-gene interactions to SNPs observed in chromosomes 7, 18 and 19.** Ratio of whole blood, adult brain, and left ventricle spatial eQTL-gene interactions to SNPs across **A)** chromosome 7, **B)** chromosome 18 and **C)** chromosome 19.


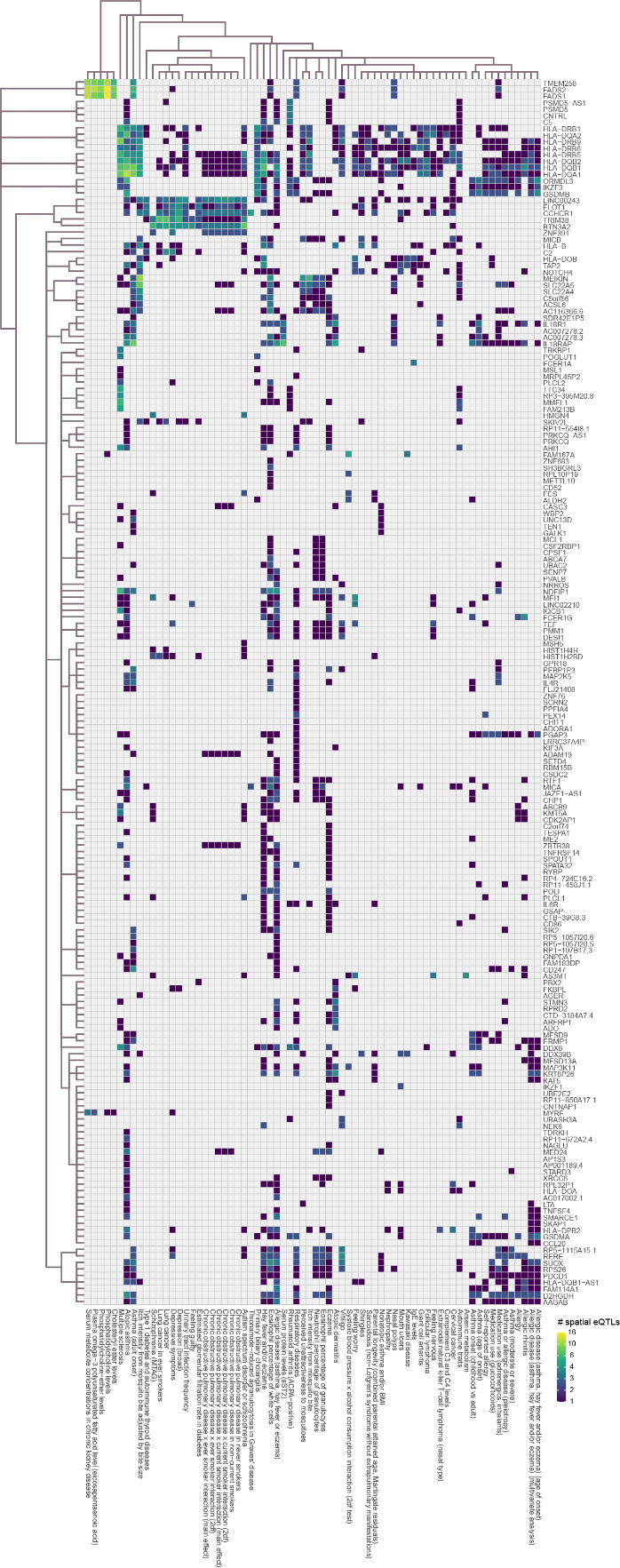


**Supplementary Figure 5. Genes regulated by asthma-associated spatial eQTLs are also regulated by spatial eQTLs associated with 70 other conditions in the blood-specific asthma GRN**. Convex biclustering of trait-eQTL-gene interactions occurring within the blood-specific asthma GRN (level 0). For a given eQTL(s), the x-axis represents the target gene, and the y-axis represents the trait associated with it.
